# FINANCIAL EXPLOITATION OF OLDER CLIENTS: EXPLORING FINANCIAL PROFESSIONALS’ ROLES AND EXPERIENCES

**DOI:** 10.1093/geroni/igad104.0885

**Published:** 2023-12-21

**Authors:** Lauren Cerino, Julie Miller, Alexa Balmuth, Adam Felts, Joseph Coughlin

**Affiliations:** Massachusetts Institute of Technology, Cambridge, Massachusetts, United States; AARP, Bethesda, Maryland, United States; Massachusetts Institute of Technology, Cambridge, Massachusetts, United States; Massachusetts Institute of Technology, Cambridge, Massachusetts, United States; Massachusetts Institute of Technology, Cambridge, Massachusetts, United States

## Abstract

Research shows that financial exploitation of older adults can be difficult to detect and underreported (Deane, 2018; Sullivan-Wilson & Jackson, 2014). Financial exploitation of older adults is both prevalent and costly, with estimated annual losses in the billions (Wood & Lichtenberg, 2017). Initiatives such as AARP’s BankSafe training program demonstrate that the financial services industry can prevent and address cases of financial exploitation (DeLiema & Deevy, 2016). But it is worth exploring how financial professionals perceive their own ability to support older adults in cases of financial exploitation. The MIT AgeLab conducted an exploratory study to examine financial professionals’ experiences with, and preparedness for, addressing financial exploitation against aging clients and their families. Data are inclusive of a sample (N=30) of financial professionals who completed an online questionnaire in April 2022. Participants’ encounters with financial exploitation in their work were wide-ranging in frequency and nature. Most participants reported that they felt well-prepared to support a client who was experiencing financial exploitation. Despite high levels of individual preparedness, participants often felt that the financial industry’s policies and services could better address elder financial exploitation. Results from this study suggest that, although financial professionals feel well-prepared as individuals to address financial exploitation, other entities—especially the financial services industry—could instill more confidence by increasing attention to the topic, implementing training, and providing resources. With additional knowledge, training, and resources, financial professionals may be able to more confidently address situations of financial exploitation.

